# Does the Maternal Gut Microbiome Influence the Outcome of Perinatal Asphyxia?

**DOI:** 10.3390/antiox14091134

**Published:** 2025-09-19

**Authors:** Vlad-Petru Morozan, Mara I. Ionescu, Carmen M. D. Zahiu, Ana Maria Catrina, Andreea Racoviță, Ana-Teodora Chirilă, Ioana-Alexandra Dogaru, Cristian Ciotei, Gratiela Gradisteanu Pircalabioru, Ana-Maria Zăgrean

**Affiliations:** 1 Division of Physiology—Neuroscience, Carol Davila University of Medicine and Pharmacy, 02001 Bucharest, Romania; carmen.zahiu@umfcd.ro (C.M.D.Z.); ana-teodora.chirila0721@stud.umfcd.ro (A.-T.C.); ioana-alexandra.dogaru0125@rez.umfcd.ro (I.-A.D.); cristian.ciotei0720@stud.umfcd.ro (C.C.); ana-maria.zagrean@umfcd.ro (A.-M.Z.); 2 Department of Plastic and Reconstructive Microsurgery, Central Military Emergency University Hospital ‘Dr. Carol Davila’, 010825 Bucharest, Romania; 3 Department of Pediatrics II, Marie Curie Emergency Children’s Hospital, 75534 Bucharest, Romania; 4 Cantacuzino National Military Medical Institute for Research and Development, 050096 Cernica, Romania; catrina.ana-maria@cantacuzino.ro; 5 Department of Dermatology, Elias University Hospital, 011461 Bucharest, Romania; andreea-stefania.racovita@rez.umfcd.ro; 6 Research Institute of the University of Bucharest, 050663 Bucharest, Romania; gratiela.gradisteanu@icub.unibuc.ro; 7 eBio-Hub Centre of Excellence in Bioengineering, National University of Science and Technology Politehnica Bucharest, 061344 Bucharest, Romania; 8 Department of Botany and Microbiology, University of Bucharest, 060101 Bucharest, Romania

**Keywords:** maternal microbiome, gut-brain axis, perinatal asphyxia, oxidative stress, neuroinflammation, neuroprotection, Nrf2 pathway, short-chain fatty acids, ferroptosis, blood-brain barrier

## Abstract

This review explores the maternal gut microbiome’s role in shaping neonatal neurodevelopmental outcomes following perinatal asphyxia (PA), a leading cause of infant mortality and disability with limited therapeutic options beyond hypothermia. We synthesized current evidence on microbiome-mediated neuroprotective mechanisms against hypoxic-ischemic brain injury. The maternal microbiome influences fetal development through bioactive metabolites (short-chain fatty acids, indole derivatives) that cross the placental barrier, bacterial antigen regulation, and infant microbiome colonization. These pathways activate multiple protective mechanisms: anti-inflammatory signaling via NF-κB suppression and regulatory T cell expansion; antioxidant defenses through Nrf2 activation; neural repair via BDNF upregulation and neurogenesis; and oxytocin system modulation. Animal models demonstrate that maternal dysbiosis from high-fat diet or antibiotics exacerbates PA-induced brain damage, increasing inflammatory markers and hippocampal injury. Conversely, probiotic supplementation, dietary fiber, and specific interventions (omega-3, resveratrol) reduce neuroinflammation and oxidative injury. Human studies link maternal dysbiosis-associated conditions (obesity, gestational diabetes) with adverse pregnancy outcomes, though direct clinical evidence for PA severity remains limited. Understanding the maternal microbiome-fetal brain axis opens therapeutic avenues, including prenatal probiotics, dietary modifications, and targeted metabolite supplementation to prevent or mitigate PA-related neurological sequelae, potentially complementing existing neuroprotective strategies.

## 1. Introduction

Perinatal Asphyxia (PA) is a medical condition in newborns caused by oxygen deprivation and hypercapnia that lasts sufficiently long to cause physical damage, particularly to the nervous system. By accounting for about half of neonatal encephalopathies in term and near-term infants, PA inflicts significant neurodevelopmental disability and ranks as the second-leading contributor to neonatal mortality [1,2,3].

Despite its high prevalence, therapeutic interventions for PA remain limited, with most offering only partial benefit [4]. While hypothermia remains the sole established treatment in clinical practice, its modest effectiveness (one out of seven treated infants avoids death or major disability) and narrow therapeutic window following birth [5,6] call for novel strategies to mitigate the consequences of hypoxic-ischemic events. Emerging evidence suggests that the maternal microbiome may be a novel and beneficial tool to address these therapeutic gaps [7,8,9].

The maternal microbiome influences foetal brain development, particularly through influencing inflammation and oxidative stress, two key drivers of hypoxic-ischemic injury in PA [7,9]. A balanced maternal microbiota creates a potent anti-inflammatory and antioxidative environment that regulates maternal immune activation and modulates microglial activity in the foetus, guiding adequate foetal neurodevelopment [10,11,12,13]. This protective milieu can buffer the developing brain against the detrimental effects of oxygen deprivation, while enhancing its neural repair capacity [10,11].

Conversely, maternal microbiota impairment, known as dysbiosis, has been linked to heightened pro-inflammatory states and disrupted metabolic signalling, exacerbating the severity of hypoxic-ischemic damage [14]. Given these findings, this narrative review aims to examine the mechanistic links and current evidence regarding the role of the maternal microbiota in PA outcomes.

## 2. Perinatal Asphyxia: Pathophysiology and Outcomes

To understand how the maternal microbiota might influence PA outcomes, it is essential to consider the pathophysiological cascade underlying this condition.

Classically, PA undergoes three distinct phases interweaved with a latent period [15]. The primary phase represents the injury itself, where low oxygen and high carbon dioxide levels trigger the chemo-reflexes, which further induce bradycardia and intense peripheral vasoconstriction to maintain perfusion of central organs [16,17]. Hypoxemia increases inflammation and oxidative stress, causes cell oedema and amplifies cell injury, increasing cellular death [18,19]. These neuronal lesions cause waves of anoxic depolarisation, which provide positive feedback in this loop and further aggravate the injuries [20,21].

Following moderate or severe hypoxic injury, a latent and apparently quiescent phase appears [22]. The higher the degree of injury, the shorter the latent period, after which the secondary phase commences with a restart of the inflammatory cascades and a continuation of the cytokine storm [5]. During this phase, trophic factors decrease, the amount of reactive oxygen species (ROS) and the mitochondrial membrane permeability increase, and intracellular calcium concentration rises, all creating a self-sustaining destructive landscape [21,23,24]. This landscape facilitates a pro-excitatory environment, which, combined with the increased glutamate resulting from neuronal loss, leads to massive depolarisation waves and seizure activity that further exacerbate neuronal loss [25,26,27].

Approximately three days after the hypoxic injury, the third phase begins [28]. Depending on the injury severity, it either tilts toward a reparatory phase, with the regeneration of damaged tissues, or, conversely, tilts toward continuing the inflammatory processes and aggravating the existing lesions [29,30,31,32].

PA produces different patterns of neurological injuries, depending on the severity and duration of oxygen deprivation, as well as the infant’s gestational age [5,33]. In preterm neonates, PA leads to a form of white matter injury called periventricular leukomalacia (PVL), which involves oligodendrocyte death and hindered myelination [34,35,36,37]. PVL presents with two distinct forms: cystic and diffuse [34,35]. In full-term neonates, acute sentinel PA damages the grey matter in the thalamus, basal ganglia, and brainstem, while repeated moderate hypoxic events lead to a “watershed” pattern, primarily affecting cortical regions [38,39]. However, depending on the context, neonates can also present multiple patterns simultaneously [5,38,40].

These lesions accompanying PA may lead to outcomes ranging from mild symptoms to neonatal hypoxic-ischemic encephalopathy (HIE), with severe hypoxic events potentially resulting in death [41]. Unfortunately, the neuronal damage often comes with dramatic long-term consequences: cerebral palsy, intellectual disability, psychiatric disorders, epilepsy, neurodevelopmental disorders (e.g., ADHD), learning disabilities, motor disabilities, etc., thus posing a great burden on the individual, as well as on society [40,41,42,43,44,45,46,47,48].

Although extensive work has focused on the pathophysiological cascades triggered by PA and the efficacy of therapeutic hypothermia and pharmacotherapy, important gaps remain in understanding why some newborns are more susceptible to brain damage than others [5,33]. The severity and persistence of PA outcomes underscore the need to investigate additional prenatal factors. In this regard, the maternal microbiome, long overlooked, has surfaced as a key player capable of influencing foetal health through immune regulation and metabolic pathways [7,9,49].

## 3. The Maternal Gut Microbiome: Composition, Functions, and Impact on Foetal Development

The maternal microbiota represents a complex consortium of microorganisms colonizing various maternal body sites, including the gastrointestinal tract, conjunctiva, airways, vagina, breast tissue, skin, and oral cavity [50]. Among these niches, the gut microbiota is the most extensively studied and undergoes significant shifts during pregnancy, driven by hormonal, metabolic, and immunological changes [51,52]. These adaptations promote enhanced energy storage and immune modulation [14,53].

In the gut, *Firmicutes* (split into several groups in the Genome Taxonomy Database) and *Bacteroidetes* (*Bacteroidota*) phyla comprise approximately 90% of the total bacterial population, with *Actinobacteria* (*Actinomycetota*), *Proteobacteria* (*Pseudomonadota*), *Verrucomicrobia*, and archaeal members such as *Euryarchaeota* accounting for the remaining 10% [51,54]. A balanced, eubiotic microbiota is characterized by diverse species, a beneficial ratio of key taxa, and vital functional capabilities, such as short-chain fatty acids (SCFAs) production, immune system education, maintenance of epithelial barrier integrity, vitamin and nutrient synthesis, and competitive exclusion of pathogens [55,56].

Despite this complexity, deviations from a balanced microbiome or dysbiosis can be categorized into three main forms: type 1, with the loss of beneficial microbes (e.g., *Lactobacillus* in the vaginal microbiome, *Bifidobacterium* in the gut); type 2, with an overgrowth of potentially harmful species (e.g., increased *Proteobacteria* during pregnancy) and type 3, with an overall reduction in microbial diversity, leading to compromised functional capacity and resilience [56].

### 3.1. Factors Influencing Microbiome Composition

Multiple factors influence the maternal microbiome during pregnancy, leading to unique microbial signatures that can impact both maternal and foetal health (Table 1). These influences create a dynamic, ever-shifting microbial landscape in the mother, which, in turn, shapes foetal development, systemic inflammation, the mother’s health, and the maturation of the foetal gut–brain axis [10,11,12,13,14,57,58,59,60,61,62,63].

### 3.2. Maternal Health Implications

**Figure 1:**
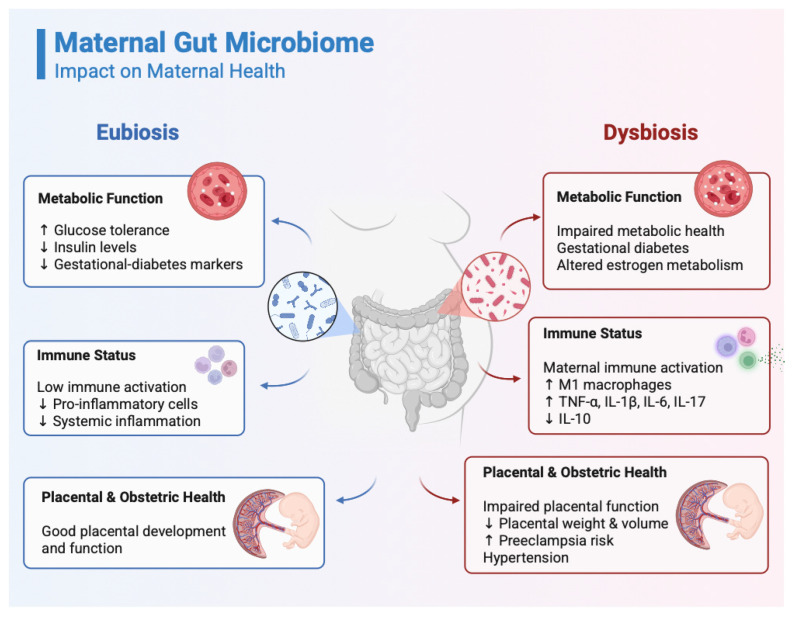
Influence of the maternal gut microbiome on the metabolic function, immune, and placental health. Left: A balanced maternal gut microbiome (eubiosis) is associated with improved glucose tolerance, reduced insulin levels, and lower markers of gestational diabetes. It is characterized by low systemic inflammation, with decreased pro-inflammatory cytokines and controlled populations of granulocytes and T-regulatory cells, as well as optimal placental development. Right: In contrast, dysbiosis is associated with impaired metabolic function (gestational diabetes, insulin resistance, altered oestrogen metabolism), heightened immune activation (elevated levels of M1 macrophages, Tumour Necrosis Factor alpha (TNF-α), Interleukin-1 beta (IL-1β), Interleukin-6 (IL-6), Interleukin-17 (IL-17); reduced levels of Interleukin-10 (IL-10); maternal immune activation), and compromised placental function (lower placental weight and volume; increased risk of preeclampsia and hypertension). Created in BioRender. Racovita, A. (2025) https://BioRender.com/75vubaj.

Maternal gut microbiome shapes maternal health throughout pregnancy (Figure 1). Dysregulated maternal gut communities are associated with elevated proinflammatory cytokines, altered oestrogen metabolism, and impaired metabolic health [84,85]. Gestational dysbiosis is associated with conditions such as preeclampsia and hypertension [13,86,87]. Probiotic administration decreases blood glucose levels, lowers gestational diabetes markers, reduces insulin levels, and improves glucose tolerance; however, some studies argue for minimal or no probiotic effect [86,88].

Beyond maternal metabolic health, gut microbes can modulate the maternal immune system by influencing populations of granulocytes and regulatory T cells (T_reg_) [13]. Diets rich in fibre, known to improve the gut microbiota, are associated with lower proportions of proinflammatory immune cells. At the same time, dysbiosis increases M1 macrophages and proinflammatory cytokines (e.g., TNF-α, IL-1β, IL-6, IL-17), and reduces anti-inflammatory cytokines such as IL-10 [13]. Moreover, dysbiosis can lead to maternal immune activation, with important consequences for both maternal and foetal health [89]. Some studies conclude that dysbiosis increases the risk of preterm birth; however, the evidence remains conflicting [90,91,92,93].

Maternal microbiota influences placental development, and dysbiosis is accompanied by lower placental weight and volume, as well as impaired maternal-foetal circulation interfaces [94]. These conditions are attenuated by SCFAs administration [94] and certain bacterial species (e.g., *Bifidobacterium breve*) [95]. Although a wave of scientific discoveries pinpointed that the placenta might present a microbiome of its own, scientific consensus tilts towards no microbiome [73,94,95,96,97,98].

### 3.3. Direct Effects on Foetal Development

**Figure 2:**
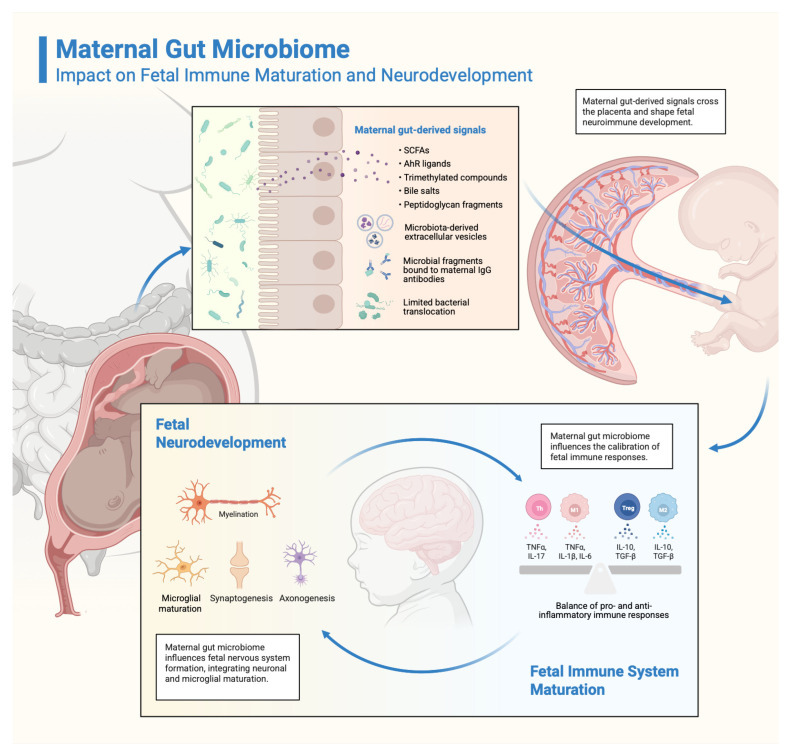
Impact of the maternal gut microbiome-derived signals on fetal immune and neurodevelopment. Bioactive microbial metabolites, namely short-chain fatty acids (SCFAs), aryl hydrocarbon receptor ligands, bile salts, trimethylated compounds, and peptidoglycan fragments, together with IgG-bound microbial antigens, microbiota-derived extracellular vesicles, and trace amounts of bacteria, cross the placental barrier and modulate fetal neuroimmune development. These signals regulate the balance between pro-inflammatory cytokines (TNF-α, IL-17, IL-1β, IL-6) and anti-inflammatory cytokines (IL-10, TGF-β), promoting microglial maturation, synaptogenesis, axonogenesis, and myelination. Created in BioRender. Racovita, A. (2025) https://BioRender.com/ye58bcx.

Aside from maternal health, pregnancy well-being, and placental development, the maternal gut microbiota influences the development of foetal immune and nervous systems, and the infant’s future metabolic health (Figure 2) [86,88].

#### 3.3.1. Immune System Maturation

From in utero to early childhood, the immune system undergoes a set of fine-tuned, sequential developmental processes [99] that evolve in parallel with the maternal gut microbiota, engaging in a bidirectional interplay [100] that shapes the immune cell populations. Dysbiosis can alter normal immune system development and can shift the balance either towards more immune reactivity with increased chances to develop autoimmune diseases or atopy, or towards a less reactive immune system, incapable of defending itself against threats [13,100,101,102,103,104,105,106,107]. For example, a maternal high-fat diet, known to induce dysbiosis, increases the expression of receptors that compromise the intestinal mucus barrier (e.g., LRRC19) [108], leading to increased bacterial translocation and heightened inflammatory state. Also, it upregulates proinflammatory RORγt-positive cells (precursors of IL-17-producing cells) [58,109] and elevates the levels of TNF-α and IL-6 [110]. In contrast, probiotics and prebiotic administration push the immune balance toward a more tolerogenic state, marked by an increase in regulatory T cells (T_regs_) and in IL-10 and TGF-β levels [111,112].

The mechanisms underlying immune system maturation involve four primary pathways: (1) microbial metabolites—SCFAs [100,107,113,114,115,116], aryl hydrocarbon receptor (AhR) ligands [10], bile salts [117], peptidoglycan fragments [118], trimethylated microbial metabolites [10], (2) IgG-bound microbial antigens [119], (3) microbiota derived extracellular vesicles [120] and (4) bacterial translocation [58].

Immune system maturation, although an autonomous process, is closely intertwined with neurodevelopment [121,122], each influencing the other’s course [123].

#### 3.3.2. Neurodevelopmental Processes

Maternal microbial communities and their metabolites impact the fetal neural circuits’ development, such as thalamocortical axons [57]. Germ-free animal models revealed altered gene expression related to myelination and axon development, highlighting the pivotal role of microbiota in neurodevelopment [10]. In addition, differentiation of GABAergic neurons appears to depend on AhR signaling [124], which is activated by microbial metabolites in the foetus.

Recently, the role of the maternal gut-fetal brain axis in fetal neurodevelopment has been increasingly considered [14]. Dysbiotic conditions lead to maternal immune activation, which has been linked to autism-like phenotypes in offspring, via IL-17-producing Th17 cells [63,89,125,126,127]. In addition, specific bacterial taxa (e.g., *Fusobacteria*) in the maternal gut have also been associated with better fine motor outcomes in children [128] and enhanced neurogenesis [129].

Maternal diet, which is known to impact microbiome, can either exacerbate [130] or mitigate [131] long-term neurodevelopmental deficits. Antibiotic use during gestation, known to induce dysbiosis, alters hippocampal development and emotional regulation in offspring [9,70], suggesting that the maternal microbiome profoundly impacts fetal brain health and development.

## 4. Neuroprotective Mechanisms Mediated by the Maternal Microbiome

**Figure 3:**
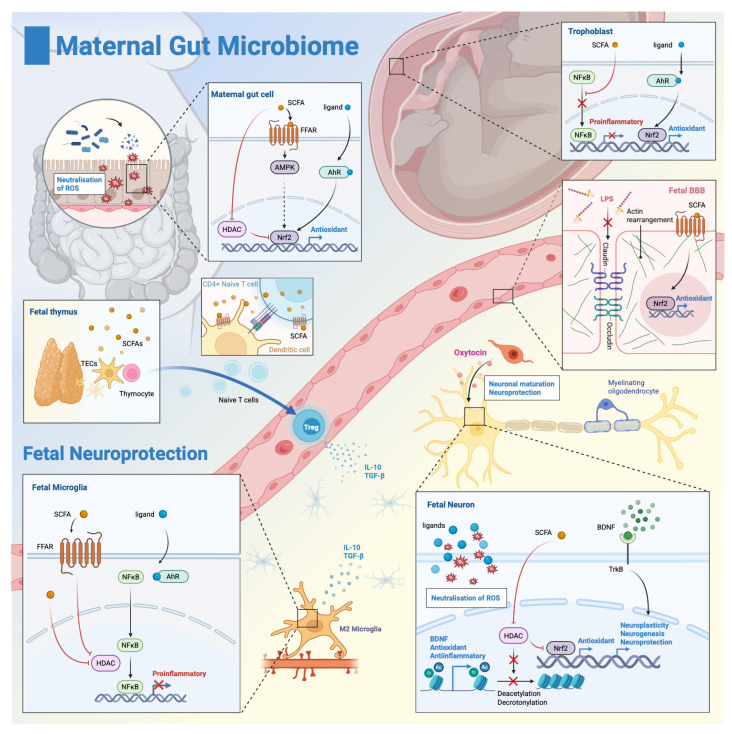
**Neuroprotective mechanisms mediated by the maternal gut microbiome**. Maternal gut microbiome-derived metabolites confer neuroprotection against perinatal hypoxic-ischemic injury through four integrated pathways spanning the maternal gut-placental-fetal brain axis. **(1)** Anti-inflammatory mechanisms: Short-chain fatty acids (SCFAs) inhibit histone deacetylase (HDAC) activity, promote M2 microglial polarization, reduce neuroinflammation, and strengthen gut-placental-blood-brain barriers. SCFAs expand regulatory T cells (T_regs_) by priming thymic epithelial cells (TECs) to select T_reg_ precursor thymocytes, dendritic cells to promote differentiation into T_regs_, and, consequently, suppress inflammatory cascades. Tryptophan-derived indole metabolites activate aryl hydrocarbon receptor (AhR), suppressing NF-κB p65 phosphorylation in microglia and decreasing IL-1β and TNF-α expression; **(2)** Antioxidant defense: eubiosis prevents ROS leaks into the circulation and contributes to upregulation of junction proteins. At the placental level, microbiota-derived metabolites directly neutralise ROS and lower oxidative stress. Through FFAR activation and Nrf2 signaling, the antioxidant capacity of the fetal BBB is increased, leading to better integrity and protection against ROS activity. In neurons, by HDAC inhibition, SCFAs induce Nrf2 activation that enhances antioxidant enzyme activity and lowers ferroptosis. AhR ligands also directly buffer some of the ROS species. Consecutive to NF-κB inhibition, M2 microglia produce less ROS, mitigating the oxidative stress; **(3)** Neural repair and neurogenesis: Microbiome metabolites maintain brain-derived neurotrophic factor (BDNF) expression through H3 crotonylation via HDAC inhibition. This promotes neurogenesis, oligodendrocyte maturation, and protective glial responses that enhance tissue preservation, myelination, and adequate pruning during the recovery phase; **(4)** Oxytocin system modulation: Specific maternal gut bacteria (particularly *Lactobacillus reuteri*) enhance hypothalamic oxytocin expression. During hypoxic stress, oxytocin facilitates the critical gamma-aminobutyric acid (GABA) switch from excitatory to inhibitory signaling, reducing excitotoxicity and providing direct neuroprotection against neuronal loss. Created in BioRender. Racovita, A. (2025) https://BioRender.com/wc5q5uf.

The response of the immature brain to metabolic deprivation conditions, such as hypoxia/ischemia, involves mechanisms still to be revealed, which could explain why some newborns walk away from the same hypoxic insult while others live with lifelong disability [132,133,134,135]. The maternal microbiome could be an important factor influencing this response: dysbiosis magnifies cytokine-driven injury [136], while eubiosis, by promoting anti-inflammatory mechanisms, redox buffering, and reparatory signals (Figure 3), blunts the secondary wave of neuronal loss that could determine whether recovery or lifelong disability prevails [137].

### 4.1. Anti-Inflammatory Mechanisms

Within hours following a hypoxic-ischemic insult, a secondary cytokine storm unleashes, with increased concentrations of IL-1β, TNF-α, and IL-6. These cytokines amplify the initial lesion size, and their levels hold predictive power for outcome forecast [138,139,140,141].

Maternal gut metabolites, mainly SCFAs and indole derivatives, can dampen that cytokine surge at several, hierarchically layered checkpoints: (i) epigenetic silencing of NF-κB inside microglia [142], (ii) tightening of gut-placenta-blood/cerebrospinal fluid barriers that stop endotoxin leakage [143,144], and (iii) prenatal expansion of T_regs_ that pre-set a tolerogenic baseline [145].

SCFAs inhibit the histone deacetylase (HDAC), which reduces the expression of inflammatory cytokines [146] and prevents NF-κB translocation, both directly, as well as through HDAC inhibition [147], thus steering microglia towards an Arg-1/IL-10 rich M2 anti-inflammatory phenotype [142,148]. In a middle cerebral artery occlusion stroke model in adult mice, butyrate, a key SCFA, almost halved infarcted volume when administered alone [149]. Moreover, in a model of neuronal damage due to cardiac arrest, butyrate attenuated the microglial inflammation through the NF-κB pathway [150] and reduced brain inflammation through activation of the JAK-STAT pathway [151]. Studies both in the adult and neonate found that the microglia respond similarly to SCFAs, by reducing NF-κB translocation, thus providing an anti-inflammatory and protective response to hypoxia [152].

SCFAs bind G-protein-coupled receptors (GPCRs) in brain endothelial and intestinal cells, providing a second layer of protection. GPCR activation increases the expression of claudin 5 and occludin in both cell types, thus tightening the barriers and limiting endotoxin accumulation in the brain [143,153]. Furthermore, SCFAs induce actin cytoskeleton rearrangements in a blood-brain barrier (BBB) in vitro model, contributing to improved BBB integrity [144]. A structurally sound BBB promotes a lower microglial activation, thus limiting the initial lesions and inflammation [154,155].

In parallel, SCFAs boost T_reg_ cells’ expansion [113,156] in the thymic and placental tissues, which lowers the fetal IL-6/IL-1β basal levels and increases the anti-inflammatory cytokine IL-10 production. SCFAs may exert these effects by directly inhibiting HDACs in naïve T cells, activating free fatty acid receptors (FFARs) on both dendritic cells and naïve T cells, and priming thymic epithelial cells to select T_reg_ cells [113,156,157].

Gut-derived indole derivatives cross the placenta and reach fetal brain tissues [158]. In animal models of ischemia, they affect microglia through AhR activation, which suppresses p65 phosphorylation and decreases IL-1β/TNF-α expression through the NF-κB pathway [159,160,161]. As shown in a stroke model, reduction of NF-κB translocation provides an anti-inflammatory and protective response to hypoxia in both adult and neonate mouse brains [152]. Experimental evidence shows that AhR-ligands interact with microglia in the fetal brain and reduce brain inflammation [162].

Epigenetic silencing of NF-κB, GPCR-driven BBB tightening, systemic T_reg_ expansion, and AhR activation form together a multilayered anti-inflammatory buffer that shields the newborn brain against PA damage.

### 4.2. Antioxidant Pathways: Maternal Gut Barrier, Placenta, Blood-Brain-Barrier, Glial Cells, and Neurons

The hypoxic injury begins with a primary burst of ROS and a larger oxidative aftershock, which result in mitochondrial failure and excitotoxicity, which together form a self-amplifying destructive loop [18]. Beyond classical apoptosis and necrosis, ferroptosis, an iron-dependent, lipid-peroxidation-driven form of regulated cell death, has emerged as an additional contributor in neonatal HIE [163,164]. Moreover, experimental data link Nrf2 activation to lower ferroptosis and improved outcomes in PA [163,165,166].

The maternal gut microbiome shapes antioxidant defences in both the mother and foetus, playing a key role in antioxidant capacity transfer to the foetus [167,168]. In mice, the maternal microbiome drives distinct metabolomics profiles in the placenta, foetal gut, and foetal brain, confirming transplacental transfer of microbial metabolites [158].

This antioxidant “defence line” spans several interconnected compartments along the maternal-foetal axis: maternal gut, placenta, epithelial barriers (BBB and placento-fetal), neurons, and glial cells.

Within the maternal gut, eubiosis sustains intestinal barrier function and neutralises ROS, preventing leakage of pro-oxidant and inflammatory molecules into the circulation [169]. A well-colonized maternal gut contributes to antioxidant protection in two complementary ways: first, gut bacteria neutralise reactive oxygen species with antioxidant enzymes—NADH peroxidase and SOD—as well as with metabolites with ROS-scavenging activity [170,171]; second, microbial metabolites increase antioxidant defence through Nrf2 signalling and upregulate tight-junction proteins [169,170,171]. SCFAs bind FFAR, inhibit HDAC, and activate AMPK, which increases Nrf2 nuclear translocation and induces antioxidant defences [170]. AhR ligands increase Nrf2 nuclear translocation and induce tight junction proteins, as well as increased enzymatic antioxidant defences [169].

In the placenta, maternal antioxidant reserves and microbiota-derived metabolites help neutralize reactive oxygen species before they reach the foetus, supporting glutathione balance and redox homeostasis [172,173], thus protecting the placenta and the foetus [174]. AhR ligands induce Nrf2 translocation, which enhances enzymatic antioxidant systems and provides antioxidant buffers themselves through interaction with IDO (indoleamine 2,3-dioxygenase) [172]. Direct placental Nrf2 activation by SCFAs in vivo remains to be demonstrated; although current evidence supports SCFA transfer and sensing, with anti-inflammatory effects through NF-κB that lower oxidative stress at the placenta [94,174].

In the foetal BBB, an increased permeability allows for more ROS increased oxidative damage in PA [175]. The microbiome regulates maturation of the BBB, controlling the expression of tight junction proteins: occludins and claudins [176]. Because SCFAs reach foetal circulation, FFAR activation and Nrf2 signalling could enhance antioxidant capacity and maintain BBB integrity [154,177].

In neurons, SCFAs protect against oxidative ischemic damage in animal models [178,179]. SCFAs inhibit neuronal HDAC [180], increase Nrf2 nuclear translocation, enhance antioxidant enzyme activity, and lower ferroptosis [181]. Additionally, they act through the BDNF-Akt3 pathway to reduce oxidative stress and apoptosis [178]. AhR ligands could exhibit direct antioxidant effects. Thus, tryptophan metabolites related to gut microbiota, which are endogenous AhR ligands, attenuated neuronal damage in an ischemic hippocampus model [182].

Glial cells, including microglia and astrocytes, further contribute by fine-tuning inflammatory responses and limiting oxidative injury, roles that have been demonstrated in ischemia and related injury models [159,165]. Mechanistically, this could involve NF-κB inhibition, which triggers M2 phenotype with lower ROS production, both through SCFA and AhR ligands [150,159], although direct evidence in PA is currently lacking.

Together, these layers form a coordinated antioxidant network that may critically influence vulnerability or resilience to oxidative injury in PA.

### 4.3. Neurogenesis, Neuroplasticity, and Neural Repair

The maternal microbiota modulates neurotrophic factor expression through pathways that lead to increased antioxidant enzymes. Short-chain fatty acids, particularly butyrate, enhance brain-derived neurotrophic factor (BDNF) expression by direct histone deacetylase inhibition and increased H3 crotonylation, which together amplify antioxidant gene transcription [183,184,185]. This SCFA-mediated BDNF upregulation activates the PI3K/AKT signaling cascade, which phosphorylates and inactivates pro-apoptotic proteins BDNF and increases antioxidant expression [186], as shown in a model of ischemia reperfusion injury [178]. In contrast, maternal high-fat diet-induced dysbiosis correlates with reduced offspring BDNF levels, along with functional impairments [187,188,189,190].

By promoting a strong anti-inflammatory phenotype in microglia, adequate synaptic pruning and development occur [191], leading to a greater neuronal reserve and improved wiring.

In dysbiotic animal models (induced through high-fat diet), butyrate improves mitochondrial function in the cerebral cortex and the synaptic fraction [192], enhances neuroplasticity [193], and shows an impressive neuroprotective effect in ischemia [142,194], all increasing the neural functional reserve. One possible mechanism underlying this protective effect could be the ability of sodium butyrate to stimulate neurogenesis, as demonstrated in rat models of perinatal asphyxia [142,195].

In addition, the maternal gut microbiome affects oligodendrocyte activation and myelination processes in the offspring’s central nervous system [196]. Through HDAC inhibition in precursor oligodendrocytes, SCFAs guide their maturation and proper myelination, creating additional resistance against PA [196]. Moreover, SCFAs reduce brain inflammation, lowering the risk of precursor oligodendrocyte disruption.

Maternal eubiosis likely primes the foetus towards a reparative phenotype during phase 3, providing direct neuroprotection through enhanced neurogenesis and neural repair, thus improving neuronal reserve and mitigating the PA effects.

### 4.4. The Maternal Microbiome, Oxytocin Signaling, and Perinatal Asphyxia—Direct Neuroprotection?

The maternal microbiome’s neuroprotective effects extend beyond direct metabolite production to include modulation of critical neuropeptide systems, particularly oxytocin (OXT) [197]. We believe this neuroendocrine pathway represents an underexplored mechanism through which the maternal gut bacteria influence fetal resilience to hypoxic injury.

Maternal gut dysbiosis, particularly from high-fat diets, depletes beneficial species like *Lactobacillus reuteri*, correlating with diminished hypothalamic OXT expression in offspring and impaired stress regulation [197]. Crucially, *L. reuteri* supplementation during pregnancy enhances maternal OXT signaling via the *vagus* nerve stimulation [198,199], with strain-specific effects not replicated by other *Lactobacillus* species [200].

The microbiome-OXT connection is particularly relevant for PA outcomes, as OXT facilitates the critical transition from excitatory to inhibitory GABA signaling in the fetal brain, a key protective mechanism against hypoxia-induced excitotoxicity [201]. In experimental PA models, OXT administration reduces neuronal loss, seizure severity, and hippocampal damage, with efficacy dependent on injury severity [202,203,204,205].

The neuroprotective capacity is enhanced by OXT’s co-release with arginine vasopressin during perinatal stress, which suppresses excessive neuronal excitability during oxygen deprivation [206,207,208]. Further, germ-free and antibiotic-exposed [209] animal models display altered OXT signaling and social deficits reminiscent of neurodevelopmental disorders, whereas offspring treatment with L. reuteri reversed maternal high-fat diet-induced social and synaptic deficits [197], suggesting that early microbial colonization is essential for normal oxytocinergic development.

Therefore, maternal dysbiosis may compromise this endogenous neuroprotective system, increase HIE severity, and disrupt OXT signalling. Microbiome-based interventions that target OXT could enhance fetal neuroprotection against PA.

## 5. The Maternal Gut Microbiome Dysbiosis and Perinatal Asphyxia Outcomes

While maternal eubiosis provides multiple layers of neuroprotection against hypoxic-ischemic injury, dysbiosis creates a contrasting scenario of increased vulnerability. Disrupted maternal microbial communities compromise the very pathways that normally shield the developing brain from oxidative damage, inflammatory cascades, and metabolic dysfunction.

This section examines the experimental evidence demonstrating how the maternal microbiome imbalance amplifies the pathophysiological processes underlying PA, transforming what might be a manageable hypoxic insult into severe, lasting neurological injury.

### 5.1. Mechanisms of Dysbiosis-Induced Neuronal Vulnerability

PA creates a storm of oxidative vulnerability that, amplified by maternal (and subsequent fetal) dysbiosis, amplifies PA’s neurological injury. This section examines how dysbiosis-derived endotoxins amplify the oxidative damage cascade specific to hypoxic-ischemic events.

As dysbiosis results in a higher lipopolysaccharide (LPS) concentration in the bloodstream [155], LPS can amplify the oxidative and proinflammatory stress [210,211], with increased ROS production, TNF-α level, and antioxidant enzymes, as shown in a model of PA, combined with LPS exposure. LPS action seems to also depend on timing [211], while even hypothermia could bring minimal benefit against a LPS sensitised neural tissue [212].

Mitochondrial dysfunction from PA is another factor of ROS and triggers multiple forms of cellular death, including ferroptosis [18]. Dysbiosis reduces bacterial metabolites that promote a tolerogenic and anti-inflammatory status, and increase oxidative stress and pro-oxidative and inflammatory cytokines such as IL-6, IL-1β, and TNF-α [181,213], thus amplifying the effect of PA.

By allowing more LPS into the bloodstream, dysbiosis affects BBB integrity [176,214,215,216], leading to enhanced neuroinflammation and, ultimately, more severe outcomes.

Lastly, by increasing pro-inflammatory cytokines and decreasing beneficial bacterial metabolites, dysbiosis transforms microglia into a pro-inflammatory phenotype with increased ROS production and neuronal damage [217,218].

### 5.2. Experimental Findings

Detrimental microbiome alterations consistently exacerbate PA-induced brain damage (Table 2). Empirical findings reveal that a high-fat gestational diet linked to gut dysbiosis heightens neuroinflammation and cellular injury in the hippocampus of rat pups subjected to PA, as shown by increased TNF-α, IL-1β, and S100B [219]. Similarly, antibiotic-induced maternal dysbiosis amplifies PA vulnerability and exacerbates brain injuries, with altered neurodevelopmental reflexes and increased hippocampal S100B [9], while neonatal antibiotic exposure reduces SCFA-producing bacteria and promotes astrocytic gliosis and microglial activation during perinatal ischemia [7].

Conversely, interventions that display neuroprotective effects have been shown to positively affect microbiome homeostasis (Table 2). Nutritional supplements, including trans-resveratrol [220], citicoline [221], and omega-3 PUFAs [222], not only provide direct neuroprotection through mechanisms such as SIRT1 activation and membrane stabilization but also promote beneficial bacteria that may protect against PA [223,224,225].

**Table 2:** Experimental Evidence Linking Microbiome Modulation to Perinatal Asphyxia (PA) Outcomes.

Intervention	Model	Primary Action	Microbiome Effect	Perinatal Asphyxia (PA) Outcomes	Key Markers	References
High-fat diet	Sprague-Dawley (SD) rats, PA P7	Metabolic dysregulation	↓ Bacteroidetes, ↑ Firmicutes/Bacteroidetes ratio	↑ Hippocampal injury	↑ TNF-α, IL-1β, S100B	[219]
Gestational antibiotics	SD rats, PA birth	Microbiome depletion ^#^	↓ Diversity, ↓ *Lactobacillus/Bifidobacterium*	↑ Brain injury, altered reflexes	↑ S100B	[9]
Neonatal antibiotics	Mice, PA	Microbiome disruption ^#^	↓ short-chain fatty acid (SCFA) producers	↑ Neuronal damage, gliosis	↑ GFAP, Iba1	[7]
Omega-3 PUFA	Mice, HI P9	Anti-inflammatory	↑ Butyrate producers ^1^*	↓ Injury volume (5 weeks)	↓ NF-κB, apoptosis	[222]
Resveratrol	SD rats, PA P7	SIRT1 activation	↑ *Lactobacillus* and *Bifidobacterium* ^2^*	↓ Hippocampal damage	↓ IL-1β, TNF-α	[220]
Citicoline	SD rats, PA P7	Membrane stabilization	Maintains homeostasis ^3^*	Preserved hippocampus	↓ Inflammation	[221]
Lactoferrin	Rats, PA	↑ Nrf2, ↓ ferroptosis	↑ *Bifidobacterium* and *Lactobacillus* ^4^*	↓ Neuronal death	↓ Ferroptosis	[226,227]
SCFAs	SD rats, PA P7	Metabolic dysregulation	Direct metabolite	↓ 30% infarct, ↑ neurogenesis	↓ IL-1β, COX-2	[194]
Probiotics ^†^	SD rats, PA birth	Microbiome depletion ^#^	↑ *Lactobacillus* and *Bifidobacterium*, ↑ SCFAs	Blood-brain barrier (BBB) protection	↓ Microglial activation	[8]

↑ increased/higher levels; ↓ decreased/lower levels; ^#^ Primary mechanism is microbiome-related; * Microbiome effects documented separately from PA studies: ^1^* [225]; ^2^* [223]; ^3^* [224]; ^4^* [227]; ^†^ *L. acidophilus* + *B. infantis*; IL-1β inflammatory model.

Lactoferrin supplementation reduces PA-induced neuronal death through Nrf2-mediated ferroptosis inhibition [226], while also promoting beneficial bacteria and inhibiting harmful ones [227].

Direct microbiome-targeted interventions provide compelling evidence for causality. SCFA administration after neonatal hypoxia reduces brain infarct volume by 30%, decreases IL-1β and COX-2 expression, and enhances neurogenesis through histone crotonylation and HDAC inhibition [142,185,194,195].

Maternal probiotic supplementation with *Lactobacillus acidophilus* and *Bifidobacterium infantis* protects blood-brain barrier integrity and reduces microglial activation even in inflammatory challenge models [8], suggesting that microbiome optimization creates resilience against multiple injury pathways.

These experimental findings demonstrate that the maternal microbiome represents a modifiable factor influencing offspring vulnerability to hypoxic-ischemic injury, with interventions showing efficacy through both direct neuroprotective mechanisms and indirect microbiome-mediated pathways.

### 5.3. Vertical Transmission and Neonatal Gut-Brain Axis Disruption

Neonates born to mothers with gut microbiome dysbiosis may vertically inherit an altered microbial community [50], potentially predisposing them to an impaired gut-brain axis function and heightened susceptibility to PA-induced neurological injury [136]. While infant gut microbiome colonization timing remains debated, most evidence indicates postnatal establishment [97], with the maternal gut microbiome serving as the primary inoculum source [50]. Multiple factors shape this vertical transmission, including maternal health status, delivery mode, breastfeeding practices, and maternal care habits [49,228,229].

This inherited dysbiosis creates a foundation of vulnerability that compounds PA pathophysiology. Because of the infant gut’s rich vascularization and the underdeveloped fetal circulatory system, gastrointestinal tissues show increased vulnerability to PA [230,231]. Animal models of global hypoxemia or mesenteric artery occlusion report upregulation of IL-1 and IL-17, gut barrier disruption, and increased bacterial translocation, reflected by higher blood LPS levels [49,232,233].

Furthermore, hypoxemic conditions consistently alter intestinal microbiome composition, with murine models showing increased Firmicutes and decreased Bacteroides and Proteobacteria populations [234,235]. This demonstrates that hypoxemia directly modifies gut microbiome composition, establishing a self-reinforcing cycle where inherited dysbiosis increases PA susceptibility while PA-induced hypoxemia further destabilizes the gut microbiome [49,236].

## 6. Therapeutic Interventions Targeting the Maternal Microbiome

Given the profound impact of the maternal gut microbiome on fetal neurodevelopment and resilience to hypoxic injury, therapeutic strategies targeting maternal microbial communities represent a promising avenue for preventing or mitigating perinatal asphyxia outcomes. These interventions range from simple dietary modifications to sophisticated microbiome-engineering approaches, each offering distinct mechanisms for enhancing neuroprotection through modulation of oxidative stress and inflammatory pathways.

### 6.1. Dietary Modifications

Maternal diet shapes its microbiome, and it also shapes the infant gut microbiome [237,238]. Despite appearing relatively straightforward, dietary interventions demonstrate significant therapeutic potential.

Dietary fibre serves as the primary substrate for SCFA production, with demonstrated anti-inflammatory effects [239,240]. High-fibre diets during pregnancy increase SCFA-producing bacteria, enhancing SCFAs levels [67,241]. Among the most studied, the Mediterranean diet, rich in vegetables, fibres, and antioxidants, seems to provide a more anti-inflammatory microbiome [242,243], with effects reverberating into early childhood, hence protection against PA [237]. A randomized control trial analysing the impact of the Mediterranean diet showed better neurodevelopment outcomes at the 2-year mark (Bayley-III) [244].

Alongside fibres, another important aspect of dietary modifications could be related to the amount of polyphenols found in the Mediterranean diet. While having an impact on the gut microbiome and on the inflammatory reverberations of the gut microbiome [224,245], polyphenols serve as antioxidants and have been found to reduce the impact of brain ischemic damage in animal models [220,246,247,248,249]. Polyphenols enhance the development of SCFAs-producing bacteria, especially *Akkermansia* and *Bifidobacterium*, and inhibit potentially harmful bacteria growth [250,251,252]. Dietary polyphenols are metabolized mainly in the colon into bioactive components, such as equol, urolithin, and protocatechuic acid, by several bacterial species (*Bifidobacterium* sp., *Lactobacillus* sp., *Bacteroides* sp., etc.) [251,253]. Through the interaction with the maternal microbiome, resveratrol, transformed into 4-hydroxyphenylacetic acid, acts through the SIRT1 pathway and prevents obesity [254].

Omega-3 fatty acid supplementation deserves special emphasis, with maternal DHA-enriched diets preventing neuronal apoptosis and reducing brain injury volume in HIE models through anti-inflammatory effects and microglial NF-κB signalling suppression [222,255], an effect that was present at 5 weeks postnatal. Alongside direct effects in animal studies, in humans, omega-3 has been associated with fewer pre-term births and better neonatal health [256]. Omega-3 fatty acids also influence maternal microbiome and the inflammatory status of the placenta and the offspring, respectively [225]. These supplements maintain the intestinal wall integrity and modulate host immune response through modulating the gut composition, promoting butyrate-producing bacteria and Bacteroidetes sp. [257].

Fermented foods such as kombucha, yogurt, and kimchi show promise for establishing a balanced maternal microbiome [258]. Furthermore, these foods have been reported to offer benefits for pregnancy overall [259]. These effects likely stem from their complex composition; while serving as a vehicle for probiotics, these foods also supply essential macronutrients (carbohydrates, proteins, fats), alongside other bioactive compounds such as prebiotics and microbial metabolites [259,260]. A review on fermented foods shows the benefits on immune system maturation, brain development and maternal health, and infant microbiota [261].

In addition, maternal administration of lactoferrin improved outcomes in PA [226] through Nrf2 signalling, increasing antioxidant buffer and reducing ferroptosis. In addition, lactoferrin modulates gut diversity and promotes beneficial bacterial growth (e.g., *Bifidobacterium* and *Lactobacillus*) [227].

Conversely, a Western-style high-fat/low-fibre dietary pattern promotes dysbiosis, elevates maternal and placental cytokine levels, and predisposes offspring to neuroinflammation [187].

### 6.2. Emerging Microbiome-Based Therapeutic Strategies

Alongside dietary and lifestyle interventions, targeted manipulation of the maternal gut microbiome through prebiotics, probiotics, postbiotics, and synbiotics represents an expanding area of research with significant implications for maternal–infant health and neurodevelopment. These interventions are distinct yet complementary, each influencing microbial composition and function via different mechanisms.

#### 6.2.1. Prebiotics: Enhancing Endogenous Microbial Metabolism

Prebiotics are defined as selectively fermentable, non-digestible food components, most notably galacto-oligosaccharides (GOS) and fructo-oligosaccharides (FOS), that stimulate the growth and activity of beneficial gut microorganisms. During pregnancy, prebiotic supplementation alters microbial fermentation patterns, leading to increased production of SCFAs such as acetate, propionate, and butyrate. These metabolites cross the placental barrier and exert systemic effects, enhancing antioxidant defense and modulating immune tolerance in the offspring [112,262]. Notably, prebiotics induce a tolerogenic immune shift by promoting regulatory B and T cells expansion and elevating acetate and butyrate concentrations, metabolites with potent anti-inflammatory functions [112,263].

Such effects may enhance fetal resilience to hypoxic-ischemic insults, although direct evidence in the context of PA is currently lacking.

#### 6.2.2. Probiotics: Augmenting Beneficial Microbial Populations

Probiotics, live microorganisms that confer health benefits when administered in adequate amounts, provide a more direct strategy to enhance maternal gut composition. Supplementation during gestation with *Lactobacillus* and *Bifidobacterium* species lowers proinflammatory cytokines, preserves the BBB, and enhances oligodendrocyte maturation following neonatal challenges [8]. Mechanistically, probiotics increase antioxidant capacity, improve immune response, suppress inflammatory cascades, and produce antimicrobial peptides that inhibit pathogenic bacteria [264,265,266,267].

Strain-specific effects have also been observed. For example, *Clostridium butyricum* enhances SCFAs production and mitigates ischemia/reperfusion injury in rodent models [179], while *Lactobacillus reuteri* modulates OXT signalling, increasing hypothalamic oxytocin expression and improving GABA-mediated neuroprotection under hypoxic stress [199,266]. In addition, probiotic interventions have reduced neuronal damage in experimental models of perinatal inflammation [8], and are clinically employed in conditions such as necrotizing enterocolitis (NEC), a pathology that can follow PA [268].

In maternal populations, probiotics have been used to reduce complications, including gestational diabetes, preeclampsia, and infection-related outcomes [111], factors that may indirectly influence PA severity if unmanaged. Nevertheless, while preclinical findings are promising, human data remain limited and inconsistent, highlighting the need for large-scale trials [269].

#### 6.2.3. Postbiotics: Delivering Microbial Metabolites Directly

Postbiotics are non-viable bacterial components or metabolites, such as SCFAs, that offer an alternative route to harness microbial benefits without live organisms. Supplementation with SCFAs has demonstrated safety and efficacy in attenuating hypoxic-ischemic brain lesions, improving BBB function, reducing oxidative stress, and suppressing inflammation in animal studies [137,154,194]. Additional evidence suggests that postbiotics may ameliorate placental dysfunction and immune dysregulation associated with dysbiosis [12,94].

#### 6.2.4. Synbiotics and Other Advanced Strategies

Synbiotics, defined as combinations of probiotics and prebiotics, represent an evolving therapeutic concept. By supplying beneficial microorganisms alongside their preferred substrates, synbiotics may synergistically enhance colonization and functional outputs. While PA-specific studies are absent, synbiotics have improved clinical outcomes in NEC and reduced allergy incidence in infants [270]. These findings suggest potential neuroprotective benefits under hypoxic stress conditions. Maternal synbiotic supplementation during gestation and lactation significantly enhances antioxidant capacity, upregulates mitochondrial function–related genes, and increases the abundance of beneficial intestinal microbiota in offspring, suggesting a synergistic benefit over probiotics alone [271].

Faecal microbiota transplantation (FMT), although still experimental in pregnancy and neonates, is another emerging modality. By transferring processed stool from healthy donors, FMT restores microbial diversity and metabolic function. Evidence supports its efficacy in metabolic syndrome and inflammatory conditions across animal and human studies [272,273,274], but its safety and utility in PA-related contexts require rigorous evaluation.

#### 6.2.5. Addressing Harmful Metabolites and Novel Pharmacological Targets

Not all microbial metabolites are beneficial. Compounds such as trimethylamine (TMA) and trimethylamine N-oxide (TMAO) are implicated in adverse cardiovascular programming and offspring hypertension [275,276]. Inhibitors like 3,3-dimethyl-1-butanol (DMB) suppress TMA/TMAO formation, mitigating these risks [276,277].

Furthermore, pharmacological targeting of the microbial-sensing pathways shows promise. FFARs agonists, such as 4-CMBT, or AhR ligands like indole-3-propionic acid (IPA), have demonstrated anti-inflammatory and neuroprotective effects in preclinical settings [159,278,279]. Such strategies, though untested in PA, may complement microbiome-directed therapies to enhance foetal resilience to hypoxia.

In summary, microbiome-based interventions, including prebiotics, probiotics, postbiotics, synbiotics, and emerging pharmacological approaches, offer exciting opportunities to improve maternal and neonatal outcomes. While animal models provide strong mechanistic evidence, clinical translation remains at an early stage. Addressing safety, timing, and strain-specific effects will be essential to realize the full potential of these therapies in preventing or mitigating the PA-related neurodevelopmental injury.

## 7. Current Challenges and Future Research Directions

Understanding the maternal microbiome’s influence on PA remains a developing area of investigation. While animal models have provided initial insights into the maternal microbiome–foetal brain axis, human research remains limited. This is compounded by methodological variability, ethical concerns, and the absence of clear clinical guidelines for microbiome-based interventions during pregnancy or neonatal care. Bridging these gaps is crucial for translating preclinical findings into effective and safe perinatal neuroprotective strategies.

### 7.1. Methodological and Scientific Gaps

Most studies to date have focused on the maternal gut microbiome, excluding other potentially influential microbial niches such as the vaginal, oral, skin, and placental microbiota. These ecosystems may play important and interactive roles in foetal immune and neurodevelopment, particularly in modulating the neonatal response to hypoxic stress. The exclusion of these sites from current studies limits our understanding of the broader maternal microbial landscape and its role in perinatal outcomes.

In addition, this review employed a narrative approach, which, while suitable for conceptual exploration, lacks the methodological rigor of systematic reviews and may not capture the full spectrum of available evidence. The majority of research investigating maternal gut microbiome influences on offspring development originates from rodent models, which enable microbiome manipulation through antibiotic depletion, gnotobiotic designs, and maternal immune activation [9,57,125]. Large animal models have validated the impact of maternal gut microbiome on infant development, both in cattle and swine [280], as well as in non-human primates [281,282,283]. Since perinatal asphyxia models are well-established in rodents, large animals, and primates, these species offer valuable platforms for investigating maternal microbiome influences on hypoxic-ischemic outcomes [142,284,285,286]. However, few studies have directly examined the interplay between maternal gut microbiome manipulation and neonatal hypoxic-ischemic injury, representing a critical research gap that these established models are well-positioned to address.

There is also a lack of longitudinal human studies examining maternal microbiome dynamics across pregnancy and their association with neonatal outcomes following PA. Without such studies, the ability to draw causal links or develop predictive microbial biomarkers remains limited.

### 7.2. Ethical and Safety Concerns

The use of microbiome-modulating therapies during pregnancy and in neonates introduces several ethical and safety concerns. Although many probiotics contain strains that are naturally present in the human gut or used in fermented foods, such as *Lactobacillus*, *Bifidobacterium*, *Streptococcus*, *Saccharomyces*, and *Bacillus*, their safety in vulnerable populations remains incompletely understood [287].

Evidence suggests that maternal probiotic use is relatively safe. A systematic review by Jarde et al. found no increased risk of miscarriage, foetal malformation, premature birth, low birth weight, or Caesarean section associated with probiotic administration during pregnancy. Therefore, targeting the maternal microbiome may represent a safer alternative to direct postnatal probiotic supplementation, particularly in preterm or at-risk neonates [90].

In contrast, neonatal probiotic use is more controversial. Case reports have documented severe adverse outcomes, including bacteraemia, fungemia, and endocarditis, in immunocompromised or preterm infants treated with probiotics [288,289,290,291], while others consider the direct administration rather beneficial [292]. In 2023, the U.S. Food and Drug Administration (FDA) issued a formal warning following reports of infections and deaths in preterm infants who received probiotics to prevent necrotizing enterocolitis [293]. These incidents underscore the need for stringent safety protocols and regulatory oversight when considering microbiota-based interventions in neonatology.

Beyond individual-level risks, public health concerns also emerge. O’Doherty et al. argue that microbial interventions may inadvertently affect others through microbial shedding or environmental transmission. For example, altering the microbiome of a pregnant individual may influence the microbial exposure of her infant, household members, or even broader community contacts. These findings highlight the importance of ethical scrutiny and the development of responsible policies around microbiome modulation, especially during critical developmental windows [294].

Taken together, these findings reinforce the need for internationally accepted standards and clinical guidelines governing the design, testing, and application of microbiome-modulating therapies in perinatal medicine.

### 7.3. Clinical Evidence and Limitations

While there are many therapeutic microbiome-targeted options, few have been studied in the context of PA and even fewer possess a straight from the bench to bedside pathway that would allow human trials soon.

Several limitations hinder clinical translation, including mechanistic uncertainty regarding how microbiome modulation leads to improved neurodevelopmental outcomes in PA, population-specific variability in maternal microbiome composition, the absence of defined timing and dosing windows for intervention during pregnancy or postpartum, and substantial regulatory and safety hurdles in ensuring maternal–foetal safety for novel therapeutics.

Furthermore, animal models, while invaluable for mechanistic insights, do not fully replicate human perinatal physiology and immune–microbiome interactions, which complicates extrapolation to clinical practice. As a result, while the therapeutic potential of microbiome-based interventions in PA is promising, progress toward clinical application will require rigorous, multi-centre studies employing standardized protocols, validated biomarkers, and long-term neurodevelopmental follow-up.

### 7.4. Future Research Recommendations

Future research should expand beyond the maternal gut microbiome to explore the contribution of other maternal microbial niches. The vaginal and placental microbiota are likely to play central roles in foetal immune education and inflammatory priming. Their influence on susceptibility to perinatal hypoxia-related brain injury remains underexplored and deserves targeted investigation.

Mechanistic studies must also evolve. Integrating high-throughput multi-omics approaches, such as metagenomics, metabolomics, transcriptomics, and immune profiling, with histological and imaging assessments of the foetal brain will allow a better understanding of how maternal microbiome signals are transduced across the placental barrier. The placenta itself is emerging as a critical intermediary in this signalling network and should be prioritized in future experimental designs.

Human clinical research must shift toward longitudinal, standardized protocols. Prospective cohort studies tracking microbiome profiles across pregnancy and associating them with neurodevelopmental outcomes in infants exposed to PA are essential. These studies should incorporate both structural imaging and behavioural assessments to evaluate the functional significance of microbial influences on neurodevelopment.

Randomized controlled trials are urgently needed to evaluate the safety and efficacy of probiotics and related interventions during pregnancy. Given current safety concerns surrounding neonatal probiotic use, maternal-targeted strategies may offer a safer route to modulate the infant microbiome indirectly. However, rigorous adverse event monitoring and stratification by maternal and infant risk factors will be crucial in any interventional study.

Finally, incorporating microbiome-based screening into routine prenatal care may enhance risk stratification for perinatal brain injury. Identifying microbial signatures predictive of adverse outcomes could support early intervention and inform the development of personalized therapeutic strategies.

In summary, the maternal microbiome represents a promising yet underutilized target for influencing the outcome of perinatal hypoxia/asphyxia. At this moment, no intervention that addresses the microbiome is currently in practice for mitigating PA effects [295]. Addressing current methodological, ethical, and clinical limitations through multidisciplinary research is essential to realize the translational potential of microbiome-based interventions in perinatal neuroprotection.

## 8. Conclusions

This narrative review shows the critical role of the maternal gut microbiome in shaping fetal immune and neurodevelopmental trajectories, with growing evidence linking its composition to neonatal vulnerability or resilience in the context of perinatal hypoxia/asphyxia. Maternal gut eubiosis may enhance brain resilience to perinatal asphyxia through potent antioxidant and anti-inflammatory stimuli that reverberate across the gut-placenta-brain axis, and through promotion of neurogenesis, neuroplasticity, and oxytocinergic pathways. Conversely, dysbiosis may exacerbate hypoxic-ischemic brain injury by priming a hyperreactive immune system, increasing oxidative stress, and negatively influencing neurodevelopmental trajectories.

The therapeutic landscape offers promising opportunities through dietary modifications, prebiotic, probiotic, and postbiotic therapeutics. However, significant challenges remain, including methodological limitations in current research, ethical considerations surrounding maternal interventions, optimal timing strategies, and the critical need for thorough safety data in pregnancy populations.

While animal studies support these associations, human data remain limited. Further research is needed to establish causality, identify key microbial mediators, and evaluate the safety and efficacy of microbiome-targeted interventions during pregnancy and the perinatal period. Bridging mechanistic insights from animal models to longitudinal human studies will be essential to translate these findings into safe, effective interventions in obstetric and neonatal practice. Therefore, harnessing the maternal microbiome as a therapeutic target could offer a promising avenue to improve perinatal neuroprotection and long-term neurodevelopmental outcomes. Ultimately, integrating maternal microbiome modulation into perinatal care—alongside established approaches such as therapeutic hypothermia—may provide a transformative strategy to reduce the burden of hypoxic-ischemic brain injury.

## Figures and Tables

**Table 1:** Factors Influencing Maternal Microbiome Composition During Pregnancy.

Factor	Effect on Microbiome	Impact Type	References
High-fat diet	↓ Bacteroidetes, ↑ Firmicutes, ↑ pro-inflammatory taxa	Dysbiotic	[64,65]
High-fiber diet	↑ short-chain fatty acids (SCFA)-producers, ↑ Bifidobacterium, ↑ diversity	Beneficial	[66,67,68]
Alcohol consumption	↓ beneficial bacteria, altered metabolic pathways	Dysbiotic	[69]
Antibiotic use	↓ diversity, loss of key commensals, ↑ resistant strains	Dysbiotic	[63,70]
Probiotic supplementation	↑ specific beneficial strains, ↑ barrier function	Beneficial	[71]
Iron supplementation	Altered Bacteroidetes to Firmicutes ratio, ↑ pathobionts	Variable	[72]
Elevated body mass index (BMI)/Obesity	↓ diversity, ↑ Firmicutes, altered SCFA production	Dysbiotic	[73,74]
Insulin resistance	Altered glucose metabolism pathways, ↓ butyrate producers	Dysbiotic	[75]
Pre-existing gastrointestinalconditions: inflammatory bowel disease (IBD)	↓ diversity, ↑ Proteobacteria, ↑ inflammatory markers	Dysbiotic	[76]
Smoking	↓ beneficial anaerobes, ↑ opportunistic pathogens	Dysbiotic	[77,78]
Regular exercise	↑ diversity, ↑ SCFA production, ↑ Akkermansia	Beneficial	[79,80]
Psychological stress	↑ Proteobacteria, ↓ Lactobacillus, altered barrier function	Dysbiotic	[13,81]
Pregnancy progression	↑ Proteobacteria (with pregnancy progression), ↓ diversity (adaptive)	Adaptive	[51,52,82]
Previous pregnancies	Enhanced microbial stability, faster adaptation	Beneficial	[83]
Maternal age	Age-dependent diversity changes, altered metabolic capacity	Variable	[51]
Residential environment	Urban vs. rural differences in diversity and composition	Variable	[51]

↑ increased/higher levels; ↓ decreased/lower levels.

## Data Availability

Not applicable.

## Abbreviations

The following abbreviations are used in this manuscript:

4-CMBT	4-Chloro-α-(1-methylethyl)-N-2-thiazolyl-benzeneacetamide
AhR	Aryl hydrocarbon receptor
AMPK	5′ adenosine monophosphate-activated protein kinase
BBB	Blood-brain barrier
BDNF	Brain-derived neurotrophic factor
BEVs	Bacterial extracellular vesicles
BMI	Body mass index
COX-2	Cyclooxygenase-2
CSF	Cerebrospinal fluid
DHA	Docosahexaenoic acid
DMB	3,3-dimethyl-1-butanol
F/B	Firmicutes/Bacteroidetes ratio
FDA	U.S. Food and Drug Administration
FFAR	Free fatty acid receptor
FFAR2	Free fatty acid receptor 2
FFAR3	Free fatty acid receptor 3
FMT	Faecal microbiota transplantation
FOS	Fructo-oligosaccharides
GABA	Gamma-aminobutyric acid
GFAP	Glial fibrillary acidic protein
GOS	Galacto-oligosaccharides
GPCRs	G protein-coupled receptors
H3	Histone 3
HDAC	Histone deacetylase
HI	Hypoxia-ischaemia
HIE	Hypoxic-ischaemic encephalopathy
Iba1	Ionised calcium-binding adapter molecule 1
IBD	Inflammatory bowel disease
IDO	Indoleamine 2,3-dioxygenase
IgG	Immunoglobulin G
IL-1β	Interleukin-1 beta
IL-6	Interleukin-6
IL-10	Interleukin-10
IL-17	Interleukin-17
IPA	Indole-3-propionic acid
JAK-STAT	Janus kinase/Signal Transducer and Activator of Transcription
LPS	Lipopolysaccharide
LRRC19	Leucine Rich Repeat Containing 19
MCAO	Middle cerebral artery occlusion
MGM	Maternal gut microbiota
MIA	Maternal immune activation
NADH	Nicotinamide adenine dinucleotide
NEC	Necrotising enterocolitis
NF-κB	Nuclear factor kappa B
Nrf2	Nuclear factor erythroid 2-related factor 2
OXT	Oxytocin
P7/P9	Postnatal day 7/9
PA	Perinatal asphyxia
PI3K/AKT	Phosphoinositide 3-kinase/Protein kinase B
PKC	Protein kinase C
PUFA	Polyunsaturated fatty acids
PVL	Periventricular leukomalacia
ROS	Reactive oxygen species
S100B	S100 calcium-binding protein B
SCFAs	Short-chain fatty acids
SD	Sprague-Dawley
SIRT1	Sirtuin 1
SOD	Superoxide dismutase
TECs	Thymic epithelial cells
TGF-β	Transforming growth factor-beta
TMA	Trimethylamine
TMAO	Trimethylamine N-oxide
TMG	Trimethylglycine
TNF-α	Tumour necrosis factor-alpha
T_regs_	Regulatory T cells
